# P-2335. Comparison of Adverse Drug Effects and Incidence of CMV Infection Between Different Dosing Regimens for Valganciclovir in the Pediatric Solid Organ Transplant Population

**DOI:** 10.1093/ofid/ofae631.2487

**Published:** 2025-01-29

**Authors:** Khaled Al Zubaidi, Anna R Huppler, Jena Jastrow, Shay Groth, Mahua Dasgupta, Ke Yan, Winfield S Waggoner

**Affiliations:** Medical College of Wisconsin, Wauwatosa, Wisconsin; Medical College of Wisconsin, Wauwatosa, Wisconsin; Children's Wisconsin, Oak Creek, Wisconsin; Childrens Wisconsin, Milwaukee, Wisconsin; MCW, Milwaukee, Wisconsin; Medical College of Wisconsin, Wauwatosa, Wisconsin; Childrens' Wisconsin, MILWAUKEE, Wisconsin

## Abstract

**Background:**

Valganciclovir has become widely used for the prevention of (CMV) infection in solid organ transplant (SOT) patients in both adult and pediatric populations. However, there is no consensus about the optimum dosing regimen in pediatric patients. Our study aims to compare the frequency of side effects and rate of CMV between two commonly used dosing regimens.

**Methods:**

In August 2020, as part of quality improvement initiative our internal SOT guideline for Valganciclovir dosing switched from weight-based (15-25 mg/kg/dose) to the manufacturer recommended dosing based on body surface area and estimated glomerular filtration rate (BSA/eGFR). We retrospectively reviewed medical records of pediatric patients (0-20 years) who underwent SOT (including liver, heart, and kidney) from 2/2015 to 2/2024. The collected data include age, transplanted organ, occurrence of abnormal liver functions (LFT) and neutropenia, and number of episodes of CMV infection during prophylaxis and 6 months post-prophylaxis. Moderate and severe neutropenia were defined as absolute neutrophil count (ANC) 500-1000 and ANC < 500, respectively.

**Results:**

116 pediatric SOT recipients were distributed almost equally between heart (32%), kidney (34%), and liver (34%) transplants. 28/116 (24%) received BSA/eGFR-based dosing. The median duration of prophylaxis was 3.9 months (IQR 2.9-6). The frequency of abnormal LFTs were similar in both dosing groups (64% in the BSA/eGFR group vs 65% in the weight-based group). Moderate neutropenia was more frequent in the BSA/eGFR group (36%) compared to the weight-based group (17%) and severe neutropenia occurred in 11% and 18%, respectively. However, these differences were not significantly different(P=0.11). CMV infection occurred in 27% of total population of patients with no significant difference in breakthrough infection on prophylaxis between the two groups (4% vs 10%, P=0.45).

**Conclusion:**

In pediatric SOT recipients, valganciclovir prophylaxis dosed based on BSA/eGFR or weight had a similar side effect profile for liver toxicity and severe neutropenia. In addition, there was no significant difference in the occurrence of CMV infections between the dosing regimens.

**Disclosures:**

All Authors: No reported disclosures

